# Application of Selected Methods to Modify Pyrolyzed Biochar for the Immobilization of Metals in Soil: A Review

**DOI:** 10.3390/ma16237342

**Published:** 2023-11-25

**Authors:** Mariusz Z. Gusiatin, Abdulmannan Rouhani

**Affiliations:** 1 Department of Environmental Biotechnology, Faculty of Geoengineering, University of Warmia and Mazury in Olsztyn, 10-719 Olsztyn, Poland; 2 Department of Environment, Faculty of Environment, Jan Evangelista Purkyně University in Ústí nad Labem, Pasteurova 15, 400 96 Ústí nad Labem, Czech Republic; a.rohani70@gmail.com

**Keywords:** heavy metals, modified biochar, soil remediation

## Abstract

Soil contamination through heavy metals (HMs) is a serious environmental problem that needs to be addressed. One of the methods of remediating soils contaminated with HMs and reducing the environmental risks associated with them is to immobilize these HMs in the soil using specific amendment(s). The use of biochar as an organic amendment can be an environmentally friendly and practically feasible option, as (i) different types of biomass can be used for biochar production, which contributes to environmental sustainability, and (ii) the functionality of biochar can be improved, enabling efficient immobilization of HMs. Effective use of biochar to immobilize HMs in soil often requires modification of pristine biochar. There are various physical, chemical, and biological methods for modifying biochar that can be used at different stages of pyrolysis, i.e., before pyrolysis, during pyrolysis, and after pyrolysis. Such methods are still being intensively developed by testing different modification approaches in single or hybrid systems and investigating their effects on the immobilization of HMs in the soil and on the properties of the remediated soil. In general, there is more information on biochar modification and its performance in HM immobilization with physical and chemical methods than with microbial methods. This review provides an overview of the main biochar modification strategies related to the pyrolysis process. In addition, recent advances in biochar modification using physical and chemical methods, biochar-based composites, and biochar modified with HM-tolerant microorganisms are presented, including the effects of these methods on biochar properties and the immobilization of HMs in soil. Since modified biochar can have some negative effects, these issues are also addressed. Finally, future directions for modified biochar research are suggested in terms of scope, scale, timeframe, and risk assessment. This review aims to popularize the in situ immobilization of HMs with modified biochar.

## 1. Introduction

The rapid expansion of cities, industries, and agriculture over the past few decades has led to a significant increase in soil contamination caused by heavy metals (HMs) [1,2]. Important concerns have been raised about the long-term effects, toxicity, and persistence of HMs in the soil [3], and these contaminants could potentially be the form of pollution that constitutes the largest risk to human health [4]. Moreover, even at low concentrations, these metals can be reactive and toxic [5]. Therefore, the development and application of efficient remediation methods for HM-contaminated soils is currently a priority for the environment. HM-contaminated soils can be remediated using physical (e.g., soil replacement and thermal desorption), chemical (e.g., vitrification, soil washing, electrokinetic remediation, and stabilization), and biological (e.g., bioremediation with bacteria and fungi and phytoremediation) methods [6,7], which each have their own benefits and limitations [8]. Technologies for physical remediation are quick and easy to implement, but they can be expensive and require large amounts of energy [9]. Chemical remediation methods efficiently remove contaminants from the environment; however, these technologies also have the potential to degrade soil characteristics via nutrient loss [10]. Biological remediation techniques are considered more environmentally friendly, but in order to be successful, they require significantly longer treatment times [11].

In view of the remediation costs and the area of soil contaminated with HM, methods based on the stabilization (immobilization) of HMs with various additives are very attractive. Thus, developing amendments that efficiently immobilize HMs in contaminated soil has been the subject of numerous studies over a substantial period of time. Biochar is increasingly recognized as a viable organic amendment for soil remediation [12,13]. It has been used to effectively remediate soil with inorganic and organic contaminants, and it is regarded as an environmentally friendly replacement for other remediation techniques [10,14]. Moreover, biochar has the potential to significantly decrease HM concentrations in plant tissues and reduce HM mobility in soil [15].

Biochar is a carbonaceous solid that is usually formed through the pyrolysis of biomass at high temperatures (300–900 °C) in low-oxygen conditions. Prominent examples of biomass used as feedstocks for producing biochar include digestates, sewage sludge, crop wastes, agricultural byproducts, energy crops, animal byproducts, and algal biomass [16,17,18,19,20]. Current studies on biomass pyrolysis have revealed that the production of biochar depends on multiple factors, including the type of biomass and its moisture content, the particle size, the ambient (flow rate and gas type), the reaction conditions (heating rate, duration, and temperature), and other factors (e.g., reactor type and catalyst) [21,22,23]. Biochar has a high degree of aromatization and is resistant to decomposition, so its use can be considered for long-term remediation. Characterization of biochar usually involves the determination of physical and chemical properties such as its porosity, specific surface area (SSA), and content of functional groups that support HM adsorption and immobilization on its interior and exterior surfaces [24,25]. Unfortunately, not every pristine biochar possesses the adsorption capacity necessary to efficiently immobilize HMs in soil. Consequently, it is crucial to develop techniques for modifying biochar that will improve its performance in HM immobilization [26]. To maximize the adsorption capacity of biochar and its performance in remediation of HM-contaminated soils, various methods have been developed to fabricate activated or modified biochars. These modified biochars are derived from pristine biochar mostly via physical and chemical methods that improve its specific surface area, porosity, cation exchange capacity, surface functional groups, pH, etc. [9,25]. For instance, modification with sodium phytate increased the capacity of biochar to adsorb Cd [27]. Similarly, the Pb sorption capacity of an H_2_O_2_-modified rape straw biochar was three times greater than that of a pristine biochar [28]. Other treatment methods, including oxidization and thiolation of biochars made from animal dung, have been proposed as strategies for better addressing HM pollution [29].

The use of modified biochar for the immobilization of HMs is a recently developed field with several knowledge gaps, primarily because the structure and sorption capabilities of biochar vary substantially depending on the raw material used for its production and the pyrolysis conditions, such as the temperature. The effect of modification is also highly dependent on the quality of the unmodified biochar; this dependence requires systematic study to identify potential patterns [29]. In recent years, a number of papers have reviewed the various techniques for modifying biochar in terms of their effects on the adsorption and removal of HMs in soil [30,31,32,33,34,35,36,37,38,39]. Most of these papers focused on biochar preparation and modification, mechanisms of adsorption and immobilization of different oxo-anionic HMs (As and Cr) and cationic HMs (Pb, Cd, Cu, etc.) in soil with modified biochars, as well as the effects of these biochars on soil properties.

However, for modified biochar to be used under real soil conditions, current knowledge needs to be systematically developed. To date, physically and chemically modified biochar has mainly been used for remediation of HM-contaminated soils, while biologically modified biochar has been more popular in the context of agricultural use of soils. Thus, the principal objective of the present review is to summarize the recent advances in biochar modification with physical, chemical, and microbial methods, with a focus on their effects on biochar functionality and the immobilization of HMs in soil. Specifically, the following topics are reviewed: (1) the status of soil contamination with HMs and strategies for soil remediation, (2) biochar modification strategies (pre-pyrolysis, co-pyrolysis, and post-pyrolysis), (3) the characterization and application of biochar modified with different methods for HM immobilization in soil, (4) the environmental impact of modified biochar, and (5) future prospects for research on the use of modified biochar in remediation of HM-contaminated soils.

## 2. HMs in Soil and Remediation of HM-Polluted Soils

Soil contamination from nonbiodegradable elements such as HMs and metalloids is a worldwide problem, and it can have devastating effects on the environment and human health [40,41].

There are an estimated 2.8 million potentially polluted sites in Europe, 19% of which need, or might need, remediation or risk-reduction measures [42]. Mining activities, smelting, municipal waste, coal combustion, smelting, traffic emissions, etc., are only some of the potential sources of HM contamination in soil [43]. Industrial and commercial activities and waste disposal and treatment have caused almost two-thirds of point-source soil pollution in Europe [44]. The main contaminants associated with these industrial activities include not only HMs (such as As, Cd, Pb, Ni, or Zn), but also mineral oils, polycyclic aromatic hydrocarbons (PAHs), halogenated and non-halogenated solvents, and polychlorinated biphenyls (PCBs). In agricultural soils, HMs, such as As, Cd, Cu, Cu, Hg, Ni, Pb, and Zn, have been detected; these contaminants come from a variety of sources, including pesticides, synthetic fertilizers, animal manure, liming materials, sewage sludge, compost, and atmospheric deposition [45]. The LUCAS Topsoil Survey, with a sampling density of 1 site/200 km^2^, found that, in the EU, agricultural land had a higher percentage of samples with concentrations above the threshold values than land subject to other uses. Out of all the samples from agricultural land (14,865 in total), 58% had HM concentrations that were above the threshold values [46]. In about 21% of agricultural soils in Europe, the levels of toxic Cd are above the regulatory threshold [45]. Moreover, contamination of agricultural soils with HMs differs between regions. Northeastern Europe and East-Central Europe were less affected by high concentrations of HMs, while most soil samples from Western Europe and the Mediterranean had concentrations of at least one kind of HM that were above the threshold [46].

Owing to their persistence, toxicity, and bioaccumulation, the threat posed by HMs has gained considerable global attention in recent decades [47]. Pollution from HMs in the soil will change the composition and quantity of organic matter in the soil, which in turn will lower the quality of crops and pose a risk to the health of humans and other organisms via the food chain [48,49]. HM pollution also endangers water and air quality, accelerates climate change, and hinders the progress of civilization towards a more sustainable future [49,50]. Human health and soil pollution are in direct conflict with each other, which is why controlling soil pollution has become a major global priority in recent years [34].

**Figure 1:**
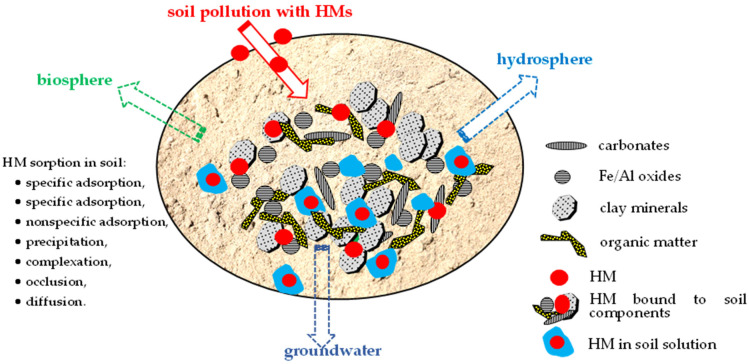
The behavior of HMs in soil and their effect on the environment.

The negative effects of HMs in the soil environment are related to their mobility, which in turn is due to the different mechanisms by which HMs interact with mineral and organic soil components (Figure 1). Soil is a complex three-phase system, comprising mineral and organic constituents, soil solution, and gases. After entering the soil, HMs that are bound to a specific soil constituent exhibit varying degrees of stability. Based on the operationally defined HM fractions in the soil, the HMs dissolved in the soil solution are the most mobile, while the HMs electrostatically bound in the exchangeable fraction can be mobilized by a slight change in the ionic strength of the soil solution. The mobility and bioavailability of HMs in the carbonate fraction increase with decreasing pH, while the availability of HMs in the reducible (Mn and Fe oxides) fraction increases with changes in redox potential. HMs bound to the oxidizable (organic matter) fraction can be mobilized after organic matter decomposition. HMs bound to the crystal lattice of soil minerals cannot be released from the soil; they are less hazardous to the environment and are considered the most stable [51]. Both HMs in the soil solution and HMs loosely bound to soil components, which can be released to the soil solution, can migrate in the soil and contaminate groundwater, as well as enter other elements of the environment, such as the biosphere and hydrosphere (Figure 1).

Based on the distribution of HMs among the various fractions, HM mobility can be evaluated using the so-called mobility factor (MF), which determines the HM content in the most mobile fractions in relation to the total HM concentration. A high MF indicates high HM mobility in soil as well as high bioavailability and bioaccessibility. According to the MF, HM mobility is classified as very high (>50%), high (31–50%), medium (11–30%), and low (1–10%). This shows that HM mobility in soil can vary depending on HM and soil type, HM concentration, and the source and time of contamination. Example values of the MF for Cd, Cr, Cu, Ni, Pb, and Zn in soils affected by various anthropogenic activities are shown in Table 1. In many contaminated sites, the mobility of commonly found HMs is high or very high. The mobility of HMs in soils affects the selection of a strategy for soil remediation and the efficiency of specific remediation methods. In general, remediation of HM-polluted soil is mainly carried out using one of two different strategies, either mobilization or immobilization.

Mobilization consists of purposefully increasing the bioavailability and mobility of HMs and metalloids in soil. It is possible to mobilize pollutants through reactions like solubilization, desorption, chelation, and complexation. These processes lead to the movement of pollutants from the solid phase to the solution phase. Consequently, this process allows for efficient and permanent HM removal from soil [52]. This approach can be carried out ex situ or in situ by applying a wide range of methods, including phytoremediation, an electrokinetic process, soil washing, etc.

**Table 1:** Examples of HM mobility (as mobility factor) in contaminated soils from different regions.

Contamination Site	HM	Total HM (mg/kg)	MF (%)	Mobility Class	Ref.
Soils around a petroleum product marketing company (Nigeria)	Pb	48.6	49	Very high	[53]
	Ni	12.3	58	Very high	
	Zn	254.2	54	Very high	
	Cd	1.8	37	High	
	Cr	35.3	41	High	
Soils from a dumping site around a Ramsar site (India)	Zn	56.3	49.5	Very high	[54]
	Cu	12.4	45.2	Very high	
Agricultural soils (China)	Cd	0.24	46	Very high	[55]
Legnica copper smelter (Poland)	Cu	8109.7	75.6	Very high	[56]
	Pb	1501.5	31.8	Medium	
	Zn	511.3	74.7	Very high	
Soils in the vicinity of a former Pb–Zn smelter in Copșa Mică (Romania)	Zn	780.0	68.7	Very high	[57]
	Cd	12.7	87.5	Very high	
	Pb	607.0	50.4	Very high	
Soils around a former Cu and blende mine (Spain)	Cu	248.0	21.2	Medium	[58]
	Zn	146.0	26.1	Medium	
Soils around a former smelter (Czech Republic)	Pb	1160.0	6.2	Low	[59]
	Zn	233.0	12.6	Medium	
	Cu	21.7.0	5.5	Low	
	Cd	5.7	56.1	Very high	

The immobilization of HMs in soils takes place via the same mechanisms that govern the interactions of HMs with inorganic and organic soil components, but these interactions are enhanced by the presence of various inorganic and organic amendments that alter certain soil properties [60]. Adsorption is among the most efficient physicochemical processes that alter the behavior and bioavailability of HMs [61]. Because of electrostatic interactions, charged solutes (ions) can bind to the charged soil surface [62]. Immobilization can be performed via the incorporation of adsorbents (such as clay minerals, zeolites, fly ash, red mud, and biochar) into the polluted soils [63]. Surface complexation reactions can also be provided by the amendment.

As a result, HMs are moved from the solution to the solid phase, becoming less susceptible to absorption by living organisms [60]. This approach involves the formation of surface complexes between HMs and functional groups on the surface of organic matter or clay minerals, such as hydroxyl, carboxyl, amino, and phenolic groups [61]. There are two distinct forms of these complexes: inner- and outer-sphere complexes. Inner-sphere complexes have no solvent molecules among the surface functional groups and ions, while outer-sphere complexes have at least one solvent molecule among them. In addition, outer-sphere complexes have lower stability than inner-sphere complexes [52,62].

Metal bioavailability can also be decreased by the precipitation of HMs. The addition of a variety of binding agents (e.g., cement, biochar, fly ash, lime, zeolite, manure, compost, chitosan, and sewage sludge) can result in the formation of a precipitate [52]. One of the most important processes that can be utilized in order to immobilize metals is the precipitation of hydroxides or sulfides within the solid matrix [63]. Precipitation with hydroxide is most efficient around pH 8 to 11 [64]. HMs in soils can also be remediated via ion exchange [65]. In this procedure, metal cations are exchanged with the surrounding phenolic groups, which ultimately results in the formation of a chelate. Inorganic zeolites that occur naturally or organic resins created in a lab could both serve as ion exchange agents [66]. However, this is a process that can be reversed. Ion exchangers can be weakly basic (tertiary and secondary amines), extremely basic (quaternary ammonium), strongly acidic (sulfonate), or weakly acidic (carboxylate), depending on the nature of the functional groups of the exchanging ions [67].

The source of soil remediation amendments can be waste, which, due to its increasing amount, requires effective and efficient waste management methods. Pyrolysis is a thermochemical recycling process that enables the recovery of gaseous and liquid hydrocarbon compounds and a carbon-rich solid (in the form of biochar) in varying proportions, depending on the type of waste and the operating parameters. These products can be used as materials in other processes and applications, so pyrolysis creates ideal conditions for a circular economy. Biochar, due to its low cost and adaptable physicochemical properties, has found potential applications in various fields, such as (i) a soil conditioner that improves soil properties and reduces the negative effects of pollutants in soil; (ii) an adsorbent for a variety of pollutants, including HMs, pharmaceuticals, PAHs, PCBs, and organic dyes; (iii) a green electrochemical material; (iv) an operational stabilizer for anaerobic digestion; and (v) a green catalyst for sustainable biorefinery and chemical conversion.

An important application of biochar is in environmental remediation. Biochar is one of numerous solidified and stabilized soil remediation materials that can be made using a wide variety of raw materials, and it is cost-effective to use and easily accessible. Due to its high specific surface area (SSA), abundance of functional groups, and excellent adsorption and complexation potential, biochar has become a critical amendment for soil in situ remediation in recent years [68]. Biochar is one of the most popular remediation materials, and its utilization is generally recognized as an efficient approach for enhancing soil quality [69]. It is a carbon-based porous substance generated by pyrolyzing biomass waste at varying temperatures and oxygen supplies [70]. However, the environmental remediation potential of pristine biochar is restricted by its relatively low sorption capacity; thus, various novel biochar modification strategies have been devised to increase its HM adsorption effectiveness [71,72].

## 3. Biochar Production and Strategies for Its Modification

Organic waste, which is the main constituent of solid biomass, has a great deal of potential for providing biochar [20]. Biomass wastes used as feedstock for biochar production can be derived from a broad range of sources, including agricultural wastes from farming, forestry, animal manure, food waste, and municipal solid waste [73]. Biochar is a carbon-rich byproduct generated via the thermal decomposition of biomass in an oxygen-deficient environment [74]. Several studies have shown that biochar is efficient at removing a wide range of pollutants [75]. Nonetheless, due to the high levels of HMs that are contained in some precursors, some materials cannot be employed in the production of biochar for the purpose of immobilizing HMs in the soil [33].

The special characteristics of biochar, particularly its large SSA, high porosity, functional groups, high capacity for cation exchange, and stability, make it suitable for a broad variety of applications. The production of biochar is quick and simple; it is safe for the environment; it can be recycled; and it is cost-effective. These are only a few of its many advantages [76]. On the other hand, it is a well-known fact that pristine biochar that has not been subjected to any pre- or post-pyrolysis modifications has a limited capacity to adsorb hazardous metals. This is because its pore structures are not fully established, and it does not have any particular binding sites that have a significant affinity for toxic metal ions [77,78]. Consequently, enhancing biochar’s adsorption capacity is crucial for its potential application in the remediation of HM contamination [79].

### 3.1. General Strategies of Biochar Modification

**Figure 2:**
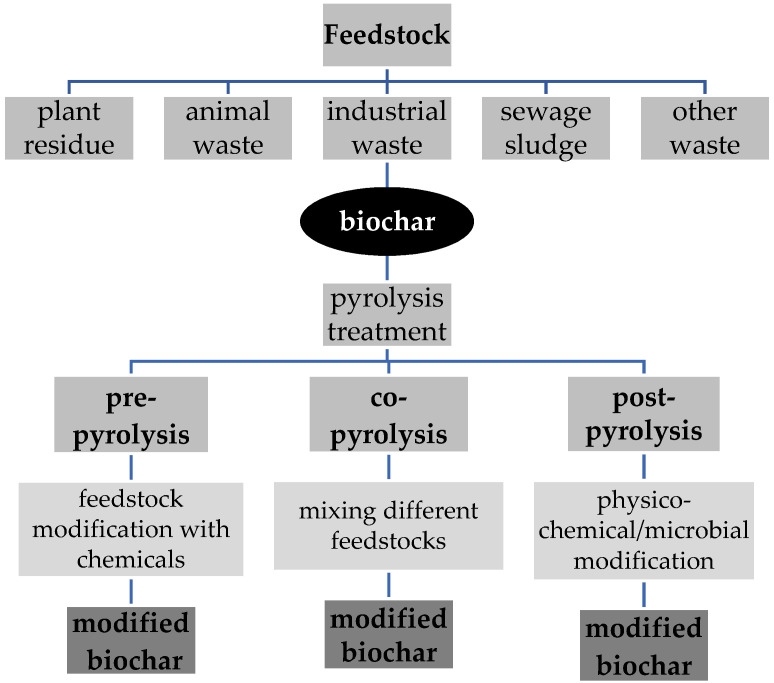
The main strategies of biochar modification at different stages of pyrolysis.

The most widely recognized raw material for creating biochar is lignocellulosic biomass, which includes waste from forests and farms [18]. This adsorbent can also be generated using non-lignocellulosic feedstocks, such as algae, animal dung, and sewage sludge [18]. Generally, the type of biomass utilized for biochar synthesis affects and is associated with the biochar’s physicochemical characteristics. The cation exchange capacity (CEC) of biochar, for instance, is affected by the type of feedstock used [18]. However, it has been shown that optimizing the pyrolysis methodology, and in particular, the modification strategy, is more effective than changing the feedstock type [80]. Hence, it has been proposed that a more efficient modification strategy be established to generate higher-quality biochar [81]. The main strategies of biochar modification are summarized in Figure 2.

#### 3.1.1. Pre-Pyrolysis

The pre-pyrolysis process involves loading feedstock with different metal compounds via impregnation with different metal salts or oxides (such as AlCl_3_, CaCl_2_, MgCl_2_, KMnO_4_, KOH, ZnCl_2_, and FeCl_3_) so that pyrolysis will form specific nano-molecules on the biochar surface, which will change its adsorption capacity by affecting its pore volume, polarity, and functional groups [82]. It has also been demonstrated that non-metallic elements (N, S, B, and P) impregnated into the feedstock prior to pyrolysis enhance its adsorption capacity [83]. Metals, including Fe and Mn, can be added to the feedstock in order to improve the biochar’s ability to bind anions, which is normally inefficient due to its negatively charged surface area. However, the applicability of this modification is determined by the type of toxin in question [82]. Another loading approach is growing seeds or cuttings with element-containing solutions, then harvesting the crop to produce biochar enriched with the desired element to use in the creation of engineered carbon [84]. Concerns about the possible release of metal compounds during biochar degradation are a significant disadvantage to this approach. Recent studies have focused on the effects of co-doping with several elements, both in metal/metal and non-metal/metal combinations. Due to their ability to decrease metal ion leaching from biochar, metal/non-metal compounds have attracted significant attention [85].

#### 3.1.2. Co-Pyrolysis

While pyrolysis conditions are crucial determinants of char properties, the development of mixed-feedstock biochars, rather than single-feedstock biochars, has recently been attempted as an additional pathway for improving biochar properties [86]. In this case, the choices between feedstocks and the relative proportion of each must be made prior to co-pyrolysis. The co-pyrolysis of a blend of feedstocks with different compositions can (i) improve biochar properties important for pollutant removal, (ii) decrease the potential environmental risk posed by biochar by diluting the content of potentially harmful compounds coming from a specific raw feedstock, and (iii) provide more efficient waste management than single-feedstock pyrolysis. For example, rice straw co-pyrolyzed with swine manure showed promise for the immobilization of HMs in soil [87]. Co-pyrolysis has gained much popularity as a method for modifying biochar’s physicochemical properties. Research in this area has employed co-pyrolysis while combining biomass and non-biomass precursors like sewage sludge, and the biochars have been characterized in terms of their agronomic value or potential for use as a soil amendment. The co-pyrolysis process fully utilizes the synergy between two starting feedstocks, which contributes to achieving higher density, greater specific surface area and pore volume, and more active functional groups. As a result, the ability of biochar to adsorb and immobilize HMs has been improved [78]. Upgraded biochar synthesized from the co-pyrolysis of biomass and polymers can be used as an adsorbent [88]. To improve the properties of biochar from co-pyrolysis, physical and chemical modification methods can be co-applied. For example, Min et al. [89] investigated the synergetic effect of ZnCl_2_ impregnation and co-pyrolysis of sewage sludge and *Camellia oleifera* shell on the surface characteristics and potential ecological risk of HMs in biochar derived from sewage sludge. The greatest benefit of this combination was the increase in biochar yield and O content and the reduction in total HM content.

#### 3.1.3. Post-Pyrolysis

Post-pyrolysis strategies involve impregnating, precipitating, and heating. Post-treatment coating involves treating biochar after pyrolysis with a solution rich in the targeted element, then evaporating or further pyrolyzing to attach the product to the surface of the biochar [90]. Biochar can also be washed in acid or alkaline solutions as a form of post-treatment [91]. The objective of acid washing is to enhance the level of acidic functional groups (carboxylic, lactonic, and phenolic) and remove contaminants from the biochar structure using either weak or strong acids (hydrochloric, sulfuric, nitric, phosphoric, oxalic, and citric acids) [71]. Based on the concentration and type of acid that is utilized, this treatment can also modify the SSA and adsorption capacity of the biochar [92]. Alkaline washing of biochar involves using solutions such as potassium hydroxide or sodium hydroxide in an attempt to improve the SSA and the amount of oxygen-containing functional groups (OCFGs) [91]. It is essential to point out that the feedstock is an important component to consider when implementing alkaline washes. For example, with some feedstocks, washing with potassium hydroxide decreased the surface area of the biochar [93]. Following pyrolysis, a process known as steam modification begins, in which free active sites are exchanged for surface hydrogen complexes (2CH) and oxide sites using gaseous water (CO) [35]. Whereas ammonia gas can be employed to add nitrogen-containing groups to the biochar, gas purging with carbon dioxide can improve the SSA and micropore structure [94]. It has also been shown that the combination of both gases improves SSA more effectively than single treatments with either gas alone [95]. In the post-pyrolysis strategy, biochar can also be modified via inoculation with microorganisms to create a biofilm with the desired properties for pollutant removal or immobilization.

Decisions about which modification strategies to perform can be based on the type of contaminant of interest and the required physical or chemical properties [35]. The functionality of biochar depending on the type of modification strategy is given in Table 2.

### 3.2. Characterization of Biochar Modified with Different Methods

The modification of biochar, including the type of method involved in the process, can be divided into three main categories: physical, chemical, and biochar-based composites [71]. In addition, microbial modification of biochar is possible.

#### 3.2.1. Physical Modification

Ball milling, exposure to microwaves, magnetic fields, steam, and gas activation are the only feasible methods for physical modification, and they require the physical disintegration of biochar. The precursor is typically pulverized into tiny particles via mechanical force and can be activated using an energy field, providing extensive adsorption sites for HMs [110]. The SSA, PV, pore structure, and surface chemical characteristics (including hydrophobicity, polarity, and functional groups) of biochar are greatly improved after undergoing physical modification. Moreover, these are simple and cost-effective procedures that require no chemical reagents [18]. In general, physical or mechanical modifications are easier to manage, cleaner, and more cost-efficient, but not as effective as chemical modifications [71].

##### Ball Milling

Ball milling is an evolving mechanochemical process that utilizes the dynamic energy of moving balls to crush and grind pristine biochar. Constant agitation and abrasion improve the SSA and change the pristine biochar’s physiochemical characteristics [111]. For example, the SSA of biochar derived from corn stover can be increased more than 3.2 times by using an optimized planetary ball mill, reaching 194 m^2^/g [112]. This process increases the content of OCFGs (e.g., carboxyl, lactone, and hydroxyl groups) [113]. An increase in acidic functional groups can lower the pH of the biochar and increase the negative surface charge. Biochar produced via ball milling can be reduced to a size of less than 100 nanometers [114]. Nano-biochar contains a broader range of aliphatic clusters and OCFGs and a lower proportion of aromatic clusters [115]. Since the ball milling process is often carried out in the air, the addition of various modifiers can lead to an increase in OCFGs [116]. Ball milling can be carried out using either chemical or physical methods. In chemical ball milling, the modification of SFGs occurs via chemical reactions that take place simultaneously with the creation of micropores and SSA modification processes during ball milling. On the other hand, physical ball milling leads to changes in particle size and SSA [117].

##### Microwave

Microwaves, which have frequencies between 300 MHz and 300 GHz, are a type of high-frequency electromagnetic wave that has many applications in the scientific and technological fields [110]. As compared to traditional pyrolysis, microwave pyrolysis requires a lower temperature and shorter reaction time in order to produce the same yield of biochar with more control over the process, more unified distributions of temperature, lower operational costs, and lower energy demands. It also makes it unnecessary to perform the pre-treatments normally used to pulverize feedstock materials [117,118]. Compared to biochar generated using traditional heating methods, biochar produced via microwave pyrolysis has a larger SSA and PV [119], and micropores are uniform and relatively clean [120]. For example, the microwave heating approach yielded biochar with a SSA of 76.3 m^2^/g, which is more than 230 times higher than the SSA (0.33 m^2^/g) of pristine biochar [121].

##### Steam/Gas Activation

Activation with gases (e.g., CO_2_, N_2_, and H_2_O) has been commonly used to promote the process of biochar production. This process converts lignocellulose into lignin and cellulose, increases the degassing rate, flushes out entrapped materials, and destroys biomass bonds, ultimately leading to the formation of crystalline biochar structures [9]. The resulting properties of the biochar are significantly influenced by the various gas components. For amorphous materials, pyrolytic degradation was faster in CO_2_ than in N_2_ [122]. In addition, the biochar produced in CO_2_ was acidic, hydrophilic, and more porous than the biochar formed in N_2_ [18,122].

Activation with H_2_O promotes the destruction of hemicellulose [123]. During steam activation, the pores of the biochar are exposed to superheated steam at temperatures between 650 and 950 °C. As a result of this process, the exchange of carbon on the surface of the biochar with the oxygen in the water molecules produces surface oxides and hydrogen [18,124]. The devolatilization and subsequent production of C-crystals in biochar are mediated by this process. The characteristic processes of steam activation involve the reciprocal transfer of oxygen from the water molecule to the surface site of the carbon, resulting in the formation of C and H_2_. Subsequently, the H_2_ that is created can potentially form complexes with the C sites. When the steam oxidizes the C surfaces, H_2_ and CO_2_ are released, which can attack the surface of the biochar and limit the gasification of the C sites. Pyrolysis during steam activation can lead to corrosion of the biochar surfaces, releasing additional syngas remaining in the biochar and generating H_2_, which ultimately increases the SSA and porosity of the biochar [71,125]. Steam activation imparts OCFGs (ether, carbonyl, carboxyl, and phenolic hydroxyl groups) to the surface of the biochar and supports the formation of meso- and macropores during the degradation of volatiles [126]. Steam-activated biochar has a higher SSA than its CO_2_-activated counterpart due to its higher OCFG content and PV [127]. Furthermore, by releasing the metal content of the biochar, it contributes to an increase in the value of the exchangeable cations [99].

##### Magnetic Modification

Biochar combined with a magnetic medium to improve solid–liquid separation has a wide range of applications in soil remediation, adsorption, and purification, and the process can be effective and simple (no modifiers or multiple processes are required) [128]. It is possible to use suitable modifying reagents (e.g., Fe_2_O_3_, FeCl_2_, FeCl_3_, etc.) together with biomass material rich in magnetic metals, including Fe and waste metal minerals, for the magnetic loading of biochar [129]. In order to achieve a good adsorption effect for a wide range of soil contaminants, the biomass can be modified accordingly before magnetic modification (e.g., via acid and alkali modification, etc.) [130]. For effective solid-phase extraction, magnetic biochar is an excellent material to use. Magnetic biochar has a high adsorption capacity for HMs and a low cost, and it can be synthesized with several possible techniques, then reused and regenerated [129].

The SSA of magnetic biochar usually varies depending on the type of magnetic species present [131] and can be very high, exceeding 2500 m^2^/g [132]. Several strategies have been developed to improve the SSA of magnetic biochar. For example, Tang et al. [133] used glacial acetic acid and nitric acid to produce a modified magnetic biochar whose SSA was three times higher than that of pristine biochar. FT-IR analysis showed that the tensile and flexural vibrational peaks of O-H were close to 3400 cm^−1^, and those of CH, CH_3_, and CH_2_ were at 2959, 2925, and 2854 cm^−1^, respectively. Furthermore, C=O bond vibration peaks were detected at 1735 cm^−1^ [134]. It has also been discovered that magnetic biochar contains persistent free radicals that play an important role in the removal of pollution. Zhong et al. [135] demonstrated that the presence of persistent free radicals in magnetic biochar contributes to the reduction in hexavalent chromium (Cr(VI)). The radicals in biochar can also break down organic pollutants by donating electrons to oxidizing agents and forming reactive oxygen species [136]. The surface charge of biochars and even the pH can be altered via magnetization. For example, Oladipo and Ifebajo [137] found that the pH of one particular biochar was 7.30, while its magnetic derivative had a pH of 8.30. The surfaces of most magnetic biochars are usually smooth, and their pore structures are highly developed. The iron oxides that are chemically bound to the biochar matrix are mostly distributed throughout the biochar, either within the pore structure or on the surface [131].

#### 3.2.2. Chemical Modification

In chemical modification, biochar is doped with specific reagent(s) to improve its properties. Chemical activation has several benefits over physical activation, including a rapid reaction time and a lower operating temperature, but it can also create much higher expenses during modification [18]. The expenses increase concomitantly with the volume and amount of chemicals used, as does the possibility that some of the chemicals may be discharged into the environment and cause secondary contamination [31].

##### Acid and Alkali Modification

Removal of impurities and enhancement of OCFGs, including −OH and −COOH, are the primary goals of acid modification. HCl, HNO_3_, H_2_O_2_, H_3_PO_4_, and many other oxidants are commonly used biochar modifiers [138]. Since acid has the potential to remove surface impurities, modifying biochar with acid can result in an increase in the porosity of the biochar as well as the SSA.

For instance, sulfuric acid and nitric acid treatments worked well to enhance the carboxyl and nitro functional groups of biochar [139]. H_3_PO_4_ may break down aromatic and aliphatic biomass structures and form phosphate/polyphosphate cross bridges that prevent contraction and shrinkage during the pore-forming process [140]. Phosphate-containing compounds have been extensively employed in recent years to remove poisonous metals from soil and aquatic ecosystems [141]. However, it has been found that HNO_3_ oxidation degrades the microporous wall, reducing surface area, owing to its corrosiveness [74].

Alkali treatment, similar to acid activation, can enhance the surface alkalinity of biochar and improve its porous structure [142]. There is some inconsistency in the findings due to the existing variety in the preparation methods and the biomass used for alkali-modified biochar. However, the surface basicity and oxygen content of biochar can be increased through alkaline activation using NaOH and KOH. Both NaOH and KOH have the potential to dissolve ash and condense organic matter (cellulose and lignin), which will make subsequent activation easier [22,110]. It is acknowledged that NaOH is preferable to KOH for carbon activation since it is less corrosive and more economical. Compared to KOH-modified biochar, the SSA and micropores of NaOH-modified biochar decrease at low pyrolysis temperatures [143]. KOH modification is reported to be especially beneficial for the formation of micropores, which in turn provide pathways for the transport of adsorbates and increase adsorption capacities [144]. Modification of biochar with Ca(OH)_2_ can effectively improve its ion exchange capacity while at the same time reducing the complexation of functional groups on its surface [145].

In general, alkali-modified biochar is more effective for removing HMs from soils than acid-modified biochar [146].

##### Oxidant Modification

Compounds with an oxidative potential, including KMnO_4_ and H_2_O_2_, improve the pore structure of biochar and raise the amount of OCFGs on its surface [118]. For example, H_2_O_2_-modified biochar made from cow manure, as reported by Wang and Liu [147], has a higher SSA and carboxyl group content but a lower ash content. However, the increase in carboxyl group content may be lower than when acid modification is used [148]. For example, compared to biochar modified with H_2_O_2_, biochar modified with HNO_3_/H_2_SO_4_ had a higher carboxyl content [149]. Furthermore, the total acidic functional group content of HNO_3_/H_2_SO_4_-modified biochar was greater than that of H_2_O_2_/KMnO_4_-modified biochar, although the total basic functional group content was lower. This suggested that oxidant treatment could be more likely to increase the number of basic functional groups in biochar [150]. Generally, deposition of MnO_x_ particles following KMnO_4_ modification of biochar often increases the interaction of the HM with the mineral components, cation-*π*, and OCFGs more than modification with other oxidants [91].

#### 3.2.3. Biochar-Based Composites

Biochar-based composites are produced via the pyrolysis of various feedstocks mixed with certain materials, mainly metals and metal oxides, nanoparticles, or carbonaceous materials (e.g., graphene oxides and carbon nanotubes).

##### Metal Ion and Metal Oxide Modification

In recent years, there has been growing interest in the research and development of metal oxides that can modify the characteristics of biochar. The primary features of biochar, such as its adsorption capacity, catalysis strength, and magnetism properties, can be improved using this modification strategy [138]. The impregnation of biochar with metals and metal oxides improves the OCFGs and pore characteristics of the biochar, resulting in primary interactions between the biochar and HM via ion exchange, surface complexation, electrostatic attraction, and cation-π bonding [24]. As an illustration, Chen et al. [70] reported that modifying biochar generated from coconut shells with MgCl_2_ improved the SSA from 1.77 m^2^/g to 79.95 m^2^/g, and the biochar also adsorbed Cd with outstanding efficiency. Biochar modification with two or more metals, which can result in the formation of different metal oxides on its surface, is receiving increased attention as a method to further improve biochar’s performance [148]. For example, the electrostatic adsorption, oxidation/reduction, and surface complexation properties of Mn-Ce-modified biochar were all greatly enhanced due to the formation of MnO_2_ and CeO_2_ on its surface [151]. The addition of ZnCl_2_ has resulted in a rise in biochar’s adsorption capacity, SSA, and porosity [152]. Modification with ZrOCl_2_ 8(H_2_O) improved the levels of zirconia and hydroxide groups on the surface of biochar, which also had a high SSA [153].

##### Nanoparticle Modification

Substances with sizes in the range of 1 to 100 nm are referred to as nanoparticles. Most nanoparticles are metal-based particles, such as zero-valent metals, metal oxides, and metal-containing nanoparticles. Other types of nanoparticles include clay minerals, graphene, activated carbon, and carbon nanotubes [154]. Compared to these other nanoparticles, metal nanoparticles exhibit greater activities towards different contaminants, notably HMs and organic pollutants, due to their higher SSA, more adsorption sites, larger surface activities, and superior mechanical characteristics [155]. As a result, metal nanoparticles have been effectively used as adsorbents, reducing agents, oxidizing agents, and catalysts for the removal of environmental pollutants [156]. However, they can lose their excellent physiochemical properties due to aggregation and passivation [157]. The use of biochar enriched with carbon can help overcome this disadvantage. The enrichment of metal nanoparticles on porous and stable biochar leads to the development of a new type of nanoparticle known as biochar-supported metal nanoparticles (MNPs@BC). This type of nanoparticle combines the advantages of biochar and metal nanomaterials. Accordingly, MNPs@BC can be created to stably anchor metal nanoparticles, modify their properties, and promote catalytic/redox processes at the biochar-metal interfaces, which ultimately maximizes the effectiveness of biochar and metal nanoparticles in environmental applications [158].

Given its physical and chemical properties, as well as its porous structure, nanobiochar is currently considered a viable option for soil improvement. It is also proving to be an efficient enzyme carrier and an excellent adsorbent. Nanobiochar particles, characterized by greater SSA, microporosity, and surface hydrophobicity, exhibit a higher absorption capacity for a variety of pollutants, including not only HMs but also herbicides, PCBs, and PAHs. Due to their higher alkalinity and pH value, they can also be used to neutralize the acidity of soils [159,160].

##### Carbonaceous Material Modification

Graphene oxides and carbon nanotubes are often used for the synthesis of composite materials that have a high bonding affinity created via functionalization with hydroxyl and carboxyl groups using chemical oxidation methods [158]. Moreover, amines and polysaccharides can also be used for modification. Slow pyrolysis was employed to pre-treat sweetgum biomass with carbon nanotubes and graphene oxide, which enhanced the SSA and capacity to adsorb Pb and Cd [161]. As reported from another batch experiment, chitosan-modified biochar proved to be more effective than untreated biochar in removing Cu, Pb, and Cd [162].

#### 3.2.4. Microbial Modification

Microbial modification of biochar is a type of bioaugmentation achieved via the immobilization of microorganisms on porous material. The immobilized microorganisms have a higher cell density and biological activity, are more resistant to environmental stresses, and are more efficient at removing pollutants [163]. Immobilization of microorganisms onto biochar is a new strategy for biochar-based materials.

Biochar is a suitable material for the immobilization of microorganisms, which can be achieved mainly through adsorption on the biochar surface, encapsulation in the biochar, and aggregation via flocculation and cross-linking [164]. Adsorption is one of the most important and useful methods for modifying biochar with microorganisms. The microbial cells are first transferred from the bulk solution to the surface of the biochar, after which they are absorbed and colonized by the biochar [163]. Biochar can also provide nutrients for the microbes, and after addition to the soil, it can change not only the soil properties but also the microbial community [109]. In particular, biochar immobilized with microorganisms is widely used to treat organic pollutants [163]. Since the microbial surface has numerous functional groups (e.g., amino, carbonyl, carboxyl, and hydroxyl groups), it can improve the capacity of biochar to sorb HMs through electrostatic attraction, ion exchange, and surface complexation [165]. Many microbial strains with high metal tolerance or adsorption capacity can be isolated from soil and used for the immobilization of HMs in soil. A positive effect on HM immobilization was achieved by combining biochar with phosphate solubilizing bacteria (PSB) or with a HM-tolerant strain of *Pseudomonas* sp. [109,166]. PSB can release phosphorus, which subsequently reacts with HMs (e.g., Pb) to form stable pyromorphite. The efficient immobilization of HMs with PSB biochar requires small biochar particles (e.g., <0.074 mm) [166]. Pseudomonas species (e.g., strain NT-2) were found to have a strong ability to promote plant growth and a high tolerance to Cu contamination. However, the toxicity of HMs and changes in the environment could affect the growth and remediation abilities of the microorganisms [167].

For biochar inoculated with microorganisms, the modified products are mainly characterized by their morphology, using qualitative techniques such as SEM, SEM/EDS, FTIR, and XRD to reveal how the microbial strains are successfully fixed on the biochar and how the functional groups and mineral composition of the biochar contribute to the immobilization effect of the HMs [109,167,168]. There are more quantitative methods for characterizing biochar modified using physicochemical methods. Therefore, a comparison of selected properties of unmodified biochar and biochar modified using physical and chemical methods is presented in Table 3; in general, the modified biochars have better properties than the pristine biochars.

## 4. Application of Modified Biochars for HM Immobilization in Soil

Recently, biochar modification has been receiving a large amount of interest due to the limitations of pristine biochar application [24]. The main physiochemical properties of pristine biochar, including organic carbon content, SSA, CEC, pH, surface functional groups, microporous structure, and inorganic minerals, are altered after modification. The application of modified biochar to soil directly alters the physical, chemical, and biological properties of the soil, which in turn affect HM immobilization. Some effects of biochar modified with physical and chemical methods and with microorganisms are briefly presented below.

### 4.1. Physically Modified Biochars

Good results in the removal and immobilization of different HMs have been achieved with magnetic biochar modified with iron-containing compounds. This method has been the subject of interest because of the benefits it provides in terms of steady adsorption performance, extremely low cost, and simplicity of regeneration [133]. In general, magnetic biochar has been shown to have higher adsorption and HM-binding capacities than nonmagnetic biochar. Impregnating biochar from waste agro-materials with magnetite/maghemite improved the sorption of Cd more than that of Pb, which implies that biochar magnetization could be more suitable for highly mobile HMs. Moreover, biochar magnetization supports stronger HM binding to biochar [96,128]. Yin et al. [182] found that magnetic biochar generated from the pyrolysis of pomelo peel using K_2_FeO_4_ as a mediator effectively absorbed Cr(VI) (209.6 mg/g). Complexation, electrostatic interaction, and reduction are the primary processes involved in Cr(VI) adsorption onto this biochar. Magnetic biochar had a maximum Cr(VI) adsorption capacity of 71.04 mg/g, and the adsorption of Cr(VI) was positively correlated with the iron concentration [183]. Application of magnetic biochar to soil from an abandoned Pb-Zn smelting site in Central China had a significant stabilizing effect on Pb and As in the soil. Magnetic biochar increased humus-like and aromatic dissolved organic matter, which had a strong binding affinity for Pb. At a biochar dosage of 2%, the immobilization of both HMs was above 90% due to efficient HM redistribution in amended soil from weak acid extraction and reducible forms to residual forms [184]. It has also been shown that magnetic biochar can immobilize HM in paddy soils. During 60 days of soil incubation with magnetic sewage sludge biochar and magnetic rice straw biochar, the bioavailabilities of Cd and Pb were reduced by 43–51% and 50–53%, respectively. Magnetic sewage sludge biochar more effectively increased the Cd and Pb content in the residual fraction than magnetic rice straw biochar, probably due to a greater tendency for HMs to co-precipitate on the crystal lattice of Fe oxides on the biochar surface [185]. On the other hand, Lu et al. [186] have reported that the temperature of pyrolysis and feedstock type can be more influential on HM immobilization than magnetization. Those authors used poultry litter and eucalyptus biomass to produce conventional and magnetic biochars at 300 and 500 °C, respectively. The conventional poultry litter biochar was found to be more effective for reducing the mobility of Cd, Pb, and Zn in soil. Interestingly, the magnetic poultry litter biochar increased plant biomass by 32%. These results suggest that magnetic biochar may have an impact on crop yield and can be considered for the management of degraded soils.

Wen et al. [187] reported that applying 3% (*w*/*w*) of FeCl_3_-modified biochar reduced the As concentration in the soil by 41.7%, in brown rice by 80.0%, and in straw by 61.5%. They also reported that biochar generated from green waste and modified with FeCl_3_ more effectively prevents rice plants from absorbing As. In another study, Yin et al. [188] also revealed that iron-modified biochar contains more oxygen functional groups than pristine biochar. The utilization of *Pennisetum sinese* Roxb and coffee ground biochars in combination with iron fertilizer considerably reduced the content of exchangeable and reducible Cd and Pb in a polluted soil, as reported by Yu et al. [189]. In addition, Fe-modified biochar was utilized to immobilize Cr in the soil; this led to a 43% decrease in the amount of available Cr [190]. Su et al. [191] reported the advantage of using nano-zero-valent Fe-loaded biochar to immobilize Cr. The modified biochar was able to immobilize 100% of Cr(VI) and 92% of total Cr within 15 days at a very low dosage (0.8%). This high immobilization efficiency was caused by the redistribution of Cr from the exchangeable form to Fe-Mn oxides and organic forms.

Studies on the use of ball-milled biochar for HM immobilization in soil are not yet numerous, but the latest studies are very promising. Xiao et al. [192] showed that the application of ball-milled bone biochar improved the growth of willow in acidic clay soils heavily polluted with multi-HMs by effectively reducing the bioavailability of Cd, Pb, Cu, and Mn and improving soil N and P fertility. Ball milling was used to grind the biochar into a micro/nanoengineered powder, which increased the SSA and exposed more surface functional groups. The stabilization effect of ball-milled biochar was significantly better than that of non-ball-milled biochar, especially at a biochar dosage of 4%, which was confirmed with the metal distribution pattern and the decrease in HM bioavailability. For example, the respective percentages of Pb in the control soil and the soil treated with ball milled biochar were 9.3% and 0.6% in the acid extractable fraction, 84.9% and 79.1% in the reducible fraction, 3.7% and 7.5% in the oxidizable fraction, and 1.9% and 12.8% in the most stable residual fraction.

A prospective modification method for biochar could be a combination of ball milling and nanoparticle loading, which could be even more beneficial for HM immobilization than using ball-milled biochar alone. A combination of ball milling (12 h) and enrichment with nanoscale P particles (at a dosage of 5%) proved to be highly efficient for immobilizing Cd and Pb in alkaline soil [193]. The nanoscale P particles, due to reactions with carbonates, alkaline minerals, and the O-containing functional groups of biochar, were converted to phosphorus oxides, phosphoric acid, and (hydro)phosphates, which reduced the soil mobility and accumulation of Cd and Pb in maize plants and alleviated the Cd and Pb stress of the plants. In addition to the immobilization of HMs, the modified biochar also had a positive effect on other soil properties that are important for comprehensive soil remediation: it improved the CEC, organic carbon content, and N and P content, and it promoted the activity of soil enzymes.

### 4.2. Chemically Modified Biochars

Very often, innovative approaches to the chemical modification of biochar are first tested based on the adsorption of a HM from an aqueous solution, and then the performance of the modified biochar in soil can be predicted, or the modified biochar can be directly used as a soil amendment. To obtain highly efficient chemically modified biochar, the modification can be carried out in several steps. For example, Zhou et al. [194] developed a unique wet pyrolysis mechanism for the in situ modification of biochar. The modification achieved functionalization of the biochar through the combined effects of H_3_PO_4_ activation, oxidation of O_2_ dissolved at the liquid/air interface, and soaking with Na_2_CO_3_ to produce CO_3_^2−^. This resulted in the production of biochar that was rich in surface functional groups and CO_3_^2−^ and exhibited a significant Pb adsorption capacity (316 mg/g) and a removal efficiency of up to 95%. Thus, biochar modified in this way can be an efficient amendment for immobilizing HMs in soil.

Many studies on the application of chemically modified biochar confirm its effectiveness for immobilizing HMs in the soil, enabling soil restoration and plant growth. Biochar from coconut shells modified with diluted HCl and ultrasounds reduced the percentage of Cd, Ni, and Zn in acid-soluble forms by 30.1%, 57.2%, and 12.7%, respectively, during a 63-day incubation after being applied to soil contaminated with multiple HMs at a dosage of 5% [195]. The improved properties of the modified biochar resulted from its improved porosity and content of functional groups. Biochar also improved the biological and biochemical properties of the soil by increasing the number of bacteria by approx. 150% [195]. Maharlouei et al. [196] also evaluated the impact of acid (H_2_SO_4_ and HNO_3_) and alkali (KOH and NaOH) modification of rice husk and almond (*Prunus dulcis* L.) soft husk biochars (pyrolyzed at 500 °C) on Cr extractability from calcareous soil. They found that the extractability of Cr was minimal with the rice husk biochar that had been treated with NaOH.

Ma et al. [197] applied a novel chemo-mechanically S-modified baby corn biochar to a Pb-contaminated (500 mg/kg) soil to evaluate tomato (*Lycopersicon esculentum* L.) growth, uptake, and soil availability of Pb. The authors pointed out the importance for Pb immobilization of the proportion of sulfur (S) in the mixture with biochar. The application of a lower proportion of elemental S (1%) and a higher proportion of biochar (2%) had the greatest effect on Pb immobilization. At this dosage, the Pb availability in the soil decreased by 37.5%, and the Pb concentration decreased in the shoots by 66.7% and in the roots by 58.3%. These results demonstrate the potential of sulfur-modified biochar for the remediation of agricultural land contaminated with HMs. S-modified biochar also had a positive effect on the reduction in Hg mobility in heavily polluted soil (1000 mg/kg). The sulfur modification of risk shell biochar increased the capacity of the biochar to adsorb Hg by about 73%. The application of this biochar at a dosage of 5% to the soil reduced the free available Hg (based on the TCLP test) by 99.3% compared to the unamended soil [198].

Through the development of insoluble HM-phosphate precipitates, phosphorus-rich biochars have the potential to reduce the levels of HMs in soil [199]. For example, husk and cornstalk biochars treated with potassium phosphate had a high efficacy of Cd immobilization [99]. In research conducted by Netherway et al. [200], P-rich biochar-treated soil contained significantly more stable Pb-phosphate and pyromorphite than the non-treated control soil. In addition, Yang et al. [201] discovered that applying P-rich biochar formed from pig carcasses to paddy soils might be an effective strategy for reducing the hazards that Pb poses to the environment and human health. Precipitation of pyromorphite-type minerals [Pb_5_(PO_4_)_3_ X; X = F^−^, Cl^−^, B^−^, or OH^−^] and the creation of Pb_3_(PO_4_)_2_ [15] are the key mechanisms by which phosphorus immobilizes Pb. Ning et al. [202] synthesized an innovative biochar from kitchen waste that showed high Pb sequestration capability after being modified with phosphates. They found that the Pb absorption with ryegrass was considerably reduced after adding P-modified biochar to the soil. The acid extractable/exchangeable fraction was clearly decreased in P-modified biochar-treated soil, which is mostly attributed to the decrease in Pb bioavailability. In another study, phosphate-loaded biochar was generated through the co-pyrolysis of phosphate and biochar by Gao et al. [101]. This biochar considerably reduced the ecological risk of Cu since the phosphate in biochar can directly be precipitated and complexed with Cu. Corn stalk biochar impregnated with K_2_HPO_4_ was applied to Pb–Zn mining soil, and the soil was incubated for 90 days. Biochar promoted the transformation of Cd, Pb, Zn, and Cu from mobile fractions (exchangeable and carbonate) to stable fractions (organic and residual) as a result of HM precipitation, complexation, and electrostatic interaction [203].

Chemically modified biochar can also be a suitable amendment in areas of manganese mining and processing. NaOH-modified grapefruit peel biochar was used as a soil amendment to determine its effect on the sorption and transport of Mn in soil [204]. The biochar used at dosages up to 10% improved the physicochemical properties of the soil (pH, TOC, and CEC) and increased Mn retention in the soil, thus preventing its migration.

Chemical modification offers a variety of options for modifying biochar and immobilizing HMs, depending on the target HM and the type and characteristics of the treated system (soil-only or soil-plant system). The latest approaches indicate that a combined chemical modification of biochar may be beneficial for the immobilization of HMs in soil. For example, Zhong et al. [205] tested cotton straw biochar and maize straw biochar simultaneously modified with KOH, K_3_PO_4_, and urea to remediate Cd-contaminated soil. The highest adsorption capacity was achieved with cotton straw biochar co-modified with K_3_PO_4_, urea, and 3M KOH. This biochar reduced the availability of Cd in soil by 52–63%, due to the redistribution of the HM into stable fractions, and reduced Cd uptake with cotton plants by over 80%.

### 4.3. Biochar-Based Composite

The application of biochar-based nanocomposites for Cr(VI) immobilization indicates the potential of biochar and nanomaterials for pollutant immobilization [30,206]. For instance, a biochar composite with nano-magnetite and siltstone with a nano-sized diameter (18.5 nm) exhibited a greater sorption capacity than the unmodified biochar (35.6 mg/g vs. 26.2 mg/g), and the incorporation of siltstone increased the stability of the composite throughout the process of thermochemical conversion [207]. In a study conducted by Ramola et al. [208], various biochar clay mineral composites were generated using discarded waste, bentonite, and calcite as the basic ingredients. Biochar composites were shown to have higher SA, porosity, and Pb adsorption capacities than unmodified biochar. A new Fe–Zn oxide composite-modified maize stover biochar was developed by Yang et al. [201]. This modified biochar has the potential to dissolve organic carbon, increase the pH and CEC of acidic soil, increase the DOC of alkaline soil, and decrease the DTPA-Cd content of alkaline and acidic soils by 23.73–52.50% and 12.77–57.45%, respectively.

The properties and structures of composite materials could be optimized by combining modification processes with physical methods, e.g., ball milling. Xie et al. [209] produced a modified biochar composite with zero-valent iron and oxalic acid via wet ball milling for the remediation of Cr(VI)-contaminated soil. The composite passivated 96.7% of Cr(VI) via adsorption, reduction, and co-precipitation mechanisms, and this state was maintained in the soil for 90 days. The high stability of Cr in the remediated soil was confirmed by its very low leaching in a TCLP test.

Herath et al. [210] used rice husk biochar, silicon fertilizer-modified rice husk biochar, and nano-montmorillonite particle-modified biochar in As-polluted paddy soils to evaluate their capability to inhibit As mobility in soil microbe-rice systems. In cultivation tests, it was shown that rice husk biochar, rice husk biochar modified with silicon fertilizer, and biochar modified with nano-montmorillonite particles all decreased the microbial-mediated discharge of As from iron minerals. Furthermore, in comparison to the control sample, the three biochar treatments considerably decreased As(III) concentrations in the rice rhizosphere by 57%, 76%, and 73%, respectively.

In order to mitigate the effects of As contamination in soil and water, Liu et al. [211] developed a nanozirconia-modified pine biochar. The results of their research revealed that the stabilization efficiency of As in the soil increased to 99.30% after 60 days with the addition of the nanozirconia-modified pine biochar, and the extractable content of As decreased from 600 to 3.1 μg/L [211].

### 4.4. Microbial-Modified Biochars

The technology of immobilizing microorganisms using biochar as a carrier and then applying it as an amendment may be promising for the remediation of soils contaminated with HMs; however, studies are scarce compared to those investigating its use for the remediation of water and wastewater [163]. Some recent studies indicate the positive effect of this amendment on the phytoremediation of HMs. Chuaphasuk and Prapagdee [212] found that low doses (0.2% *w*/*w*) of biochar with immobilized Cd-resistant bacteria (*Arthrobacter* sp. TM6 or *Micrococcus* sp. MU1) enhanced Cd phytoextraction by increasing Cd accumulation in the roots and shoots of *Chlorophytum laxum* R.Br. The application of biochar inoculated with microorganisms can restore soil function. Tu et al. [109] demonstrated that corn biochar loaded with strain NT-2 of Pseudomonas sp. increased soil pH to the neutral-alkaline range, increased the stability of Cd and Cu by redistributing HMs from exchangeable and carbonate fractions to the residual fraction, and increased the relative abundance of the genus Pseudomonas, but slightly decreased microbial diversity. Biochar modified with ureolytic bacteria that degrade urea and produce carbonate ions, which are important for the immobilization of HMs in the soil, has a similar effect on soil functions as biochar loaded with strain NT-2. Xu et al. [213] directly used rice straw biochar (1%, *w*/*w*) together with the strain *Sporosarcina pasteurii* (10 mL bacterial solution) to immobilize Cd in soil. The efficiency of Cd immobilization was almost two times higher compared to unmodified biochar. The Cd in the amended soil was precipitated in the form of octavite (CdCO_3_). In addition, soil fertility and bacterial diversity were significantly improved. Microbial modification of biochar can be considered an environmentally friendly and sustainable method for remediation of polluted soils, especially agricultural soils, and should be further developed in the near future.

## 5. Possible Negative Environmental Effects of the Use of Modified Biochar in Soil Remediation

Different modified biochars have been produced to address various soil contaminants and ecological requirements. Modified biochar plays an important role in the immobilization of HMs by altering many physical and chemical properties of pristine biochar, but it also alters soil quality. So far, most studies have focused on the development of modification processes for biochar upgrading to achieve the highest efficiency of HM immobilization and on analyzing the main mechanisms involved in this process. Despite the many benefits of modified biochar, the potential environmental hazards it may pose cannot be ignored and must also be considered. The risks associated with the use of modified biochar should be analyzed, as this is crucial for the comprehensive development of engineered biochars and their application in biochar-based soil remediation technologies.

Although many modified biochars have positive effects on physical and hydraulic soil properties as well as on soil organic matter accumulation, chemically modified biochars can alter soil salinity by increasing electrical conductivity, especially when many reagents or multi-step chemical processes are used for their modification. In addition, the activation procedure for chemically modified biochar can involve the application of some chemicals that can be toxic; therefore, it is critical to evaluate the stability of this modified biochar to prevent secondary contamination [34]. In contrast to reports of the immobilization of HMs with biochar, there are several examples of biochar promoting the leaching of HMs to varying degrees [33].

Small-sized biochar particles obtained through physical modification can change soil pore structure and diminish infiltration [214]. On the other hand, biochar nanoparticles have the ability to move down the soil profile and penetrate the groundwater system. Biochar nanoparticle mobility in the soil increased with decreasing ionic strength. Moreover, humic acid increases the mobility of biochar nanoparticles, which may be discharged to groundwater in soils enriched with organic matter [215].

It has been shown that modified components in modified biochar can affect soil microbial activities. For example, the presence of sulfur in biochar facilitated the development of *Thiobacillus*, which lowers soil pH, increases the reduction potential of oxidation, and ultimately promotes Pb mobility [216]. There have been examples where biochar contributed to an increase in Cd accumulation in plants. For instance, in order to remediate soil polluted with As, Cd, and Pb, Wen et al. [187] utilized biochar that had been modified using Fe. In that study, after the use of Fe-modified biochar, the concentration of As in straw and brown rice was reduced, while the concentrations of Cd and Pb increased. Since the Fe-modified biochar produced by the authors was acidic (pH of 4.41), the addition of this biochar increased the cation concentration in the soil and increased the release of Cd and Pb from the soil.

## 6. Conclusions and Future Perspectives

It is possible to modify biochar either before, during, or after the pyrolysis process. Physical, chemical, and microbial modifications are currently among the most commonly used techniques for upgrading biochar properties to immobilize HM in contaminated soils. Physical modification is clean and easier to control, while chemical modification creates biochar with good functionality. Biochar modified with HM-tolerant microorganisms can achieve good effects in HM immobilization and soil restoration. Modifying biochar via various strategies and methods can have distinct effects on its physical and chemical characteristics (usually increasing the surface area, pore volume, cation exchange capacity, and content of functional groups), as well as its efficiency for absorbing and immobilizing HMs.

The effectiveness of modified biochar in immobilizing a HM depends not only on the type of feedstock and its pyrolysis conditions, but also on the method of modification and the type and properties of the modifier. The literature shows that there are many examples of biochar modified using different protocols. Among them, chemically modified biochar products predominate. This is because it seems to be easier to manipulate the properties of biochar for the immobilization of particular HMs with chemical modification than with other methods. However, more efforts should be made to develop hybrid methods of biochar modification to make the synthesis of modified biochar cheaper and more environmentally friendly. These advances could popularize and add value to the practical application of modified biochar on a field scale.

Most results on the immobilization of HM with modified biochars have come from controlled, short-term laboratory trials (1–3 months on average). Even though soils are very often co-contaminated with HMs, the immobilization efficiency has often been determined for only one or two HMs. Therefore, further investigations should focus on the use of modified biochars and real HM-contaminated soils or be performed directly at field scale under a wide range of environmental conditions (e.g., temperature and moisture regimes), and longer-term remediation should be investigated. These studies would be important for a better understanding of not only HM immobilization but also of the activity and stability of the biochar after HM absorption.

Moreover, the potential environmental risk of biochar modified with different methods is not monitored in amended soil, as the main objective of the research has been the efficiency of the immobilization of HMs in soil. In order to determine the negative effects of modified biochars in remediated soils, environmental (e.g., leaching of HM and other pollutants) and human risk assessments should be included in the remedial process.

## Figures and Tables

**Table 2:** Examples of modification protocols for biochars and their effect on biochar functionality.

Strategy of Modification	Type of Biomass	Type of Process/Modifier	Conditions	Main Effects	Ref.
Pre-pyrolysis	orange peel	Co-precipitation of Fe^3+^/Fe^2+^ (magnetite)	pH 10, 30 min, stirring	Decreased SSA (by 482)/increased APR (2.0); high sorption capability	[96]
	pine wood	Hematite treatment ɣ-Fe_2_O_3_ (maghemite)	120 min, mixed	Slightly decreased SSA (16.5); strong magnetic property	[97]
	rice straw	Calcium-based magnetic biochar: Fe^3+^/Fe^2+^ and CaCO_3_	400 °C, 2 h, ground through a 0.15 mm sieve and stored	Slightly decreased CEC (7.8); increase in soil pH (by 0.19)/complexation on iron oxide surface	[98]
	camphor wood chips, bamboo offcut, cornstalk, rice husk	Phosphorus-modified biochars: K_3_PO_4_	550 °C, 2 h.	OCFGs, P-O groups, and P-C groups were introduced to biochar surface; no significant change in SSA and PV	[99]
Co-pyrolysis	rice straw (RS) sewage sludge (SS)	Pyrolysis in a muffle furnace at different mass ratios of RS:SS (1:0, 3:1, 2:1, 1:1, 1:2, 1:3, and 0:1)	400 °C 2 h	The optimum RS/SS ratio of 1:2, improved biochar yield, decreased TOC content, and increased biochar pH and surface porosity. Cd bioavailability in soil decreased by 67–86%	[100]
	rap estraw (RS), orthophosphate (P)	Pyrolysis of RS and P at mass ratio of 1:0.456	500 °C 2 h	Improved pH of biochar and number of functional groups. Improved metal immobilization in soil, especially Pb and Cu	[101]
	microplastics (MPs) sewage sludge (SS)	Slow pyrolysis for different dosage ratios of MPs in the mixture (5–15%) Flash pyrolysis for different dosage ratios of MPs in the mixture (5–15%)	500 °C 1 h 700 °C 7 min	Biochar more effective from slow than fast pyrolysis due to larger molar O/C and O+N/C ratios, enhanced surface area and PV, and the presence of spherical- or ellipsoidal-shaped macro/mesopores Transformation of Cr and Pb into residual fraction in soil with biochar containing 10 and 15% of MPs	[102]
	corn stover (CS) industrial coal ash (CA)	Horizontal tube reactor, CS and CA mixed at mass ratio 1:1	450 °C 1 h	Significantly enhanced immobilization of Pb (by 71%) and Cd (by 45%) in soil due to increased pH value, surface functionality, and surface negative charge	[103]
Post-pyrolysis	paper mill sludge	Iron solution treatment (+ surfactant + reducing agent (Zero Valent Iron))	30 min, vigorous stirring	Increased SSA (by 34)/slight decrease in PV; low iron leaching from the biochar matrix	[104]
	walnut shells	Ball milling Fe_3_O_4_	Ball milled, 6 h, 550 rpm	Increased SSA (by 334)/PV (by 0.50); a fast and high adsorption rate	[105]
	pine sawdust	Oxidative hydrolysis Fe_3_O_4_	90 °C N_2_ protection	Decreased SSA (by 172)/slight increase in PV (by 0.06); high saturation magnetization; and adsorption was exothermic with physisorption	[106]
	wheat straw	Sulfur-modified biochar: NaOH/CS_2_	NaOH and CS_2_ were mixed at a ratio of 2:3; stirred at 40 °C for 16 h	Slight increase in SSA (by 1.7) and PV (by 0.003)	[107]
	corn straw	KMnO_4_/Fe(NO_3_)_3_	600 °C for 0.5 h, KmnO_4_:Fe(NO_3_)_3_ weight ratio of 25:4:1.	Increased SSA (by 147); increased soil redox potential; and improved soil enzyme activities	[108]
	maize straw	Biochar with *Pseudomonas frederiksbergensis* strain NT-2	Cultivation of the strain, blending with biochar at 1:3 ratio, incubation (8 h)	Microbial cell aggregation on biochar surface, the presence of extracellular polymeric substances around the cells, and increased HM stability in soil	[109]

Abbreviations: SSA, specific surface area (m^2^/g); APR, average pore radius (nm); CEC, cation exchange capacity (cmol/kg); and PV, pore volume (cm^3^/g).

**Table 3:** Effect of physical and chemical modification methods on selected properties of different biochars.

No.	Biochar	Description	Selected Properties	Ref.
Physical modification				
1.	pristine	pine, pyrolysis temp. 600 °C	SSA: 197; PV: 0.078; AC: 0.15	[169]
	modified	ball-milled biochar	SSA: 337; PV: 0.078; AC: 0.90	
2.	pristine	woodchips, pyrolysis temp. 300 °C	SSA: 1.21; PV: 0.003; PS: 17.80	[170]
	modified	ball-milled biochar	SSA: 7.92; PV: 0.034; PS: 21.50	
3.	pristine	hickory wood chips, pyrolysis temp. 600 °C	SSA: 214.62; PV: 0.009; PS: 1.14	[171]
	modified	ball- milled biochar	SSA: 286.45; PV: 0.100; PS: 0.587	
4.	pristine	spruce and fir, pyrolysis temp. 475 °C,	SSA: 50; PV: 0.03; PS: 1.5; pH: 6.6; AS: 1.2	[172]
	modified	steam activation	SSA: 1025; PV: 0.77; PS: 3.0; pH: 10.3; AS: 11.3	
5.	pristine	food waste, pyrolysis temp. <500 °C	SSA: 3.97; PV: 0.007; PS: 7.30	[173]
	modified	biochar-assisted high-solid anaerobic digestion coupled with steam gasification	SSA: 160.66; PV: 0.213; PS: 5.63	
Chemical modification				
6.	pristine	waste palm shell, pyrolysis temp. 600 °C	SSA: 185.8; VM: 20.6; AS: 3.3	[174]
	modified	KOH activation	SSA: 301.1; VM: 17.5; AS: 3.0	
7.	pristine	rice straw, pyrolysis temp. 500 °C	SSA: 1.16; PV: 0.0053; PS: 18.14; AS:16.38	[130]
	modified	alkali-modified biochar (NaOH)	SSA: 260.59; PV: 0.2310; PS: 3.55; AS: 22.71%	
8.	pristine	cedar, pyrolysis temp. 300 °C	SSA: 533; PV: 0.095; AS: 22.5%; pH: 6.3	[175]
	modified	iron-modified magnetic biochar	SSA: 370; PV: 0.077; Ash: 26.9%; pH: 9.7	
9.	pristine	rice husk, pyrolysis temp. 400 °C	SSA: 4.01; PV: 0.0103; PS: 13.31–39.54	[176]
	modified	ZnO nanoparticles	SSA: 13.84; PV: 0.0155; PS: 4.51–17.65	
10.	pristine	wheat straw, pyrolysis temp.	SSA: 2.94; PV: 0.0017; AS: 16.12	[107]
	modified	sulfur-modified biochar	SSA: 4.64; PV: 0.0042; AS: 20.80	
11.	pristine	spent coffee grounds, pyrolysis temp. 800 °C	SSA: 4.0; PV: 0.006; PS: 5.818; AS: 12.6	[177]
	modified	alkali-modified biochar	SSA: 427.5; PV: 0.006; PS: 3.113; AS: 15.4	
12.	pristine	rice hull, pyrolysis temp. 550 °C	SSA: 6.2; PV: 0.014; PS: 8.839; AC: 1.638	[178]
	modified	acid-modified biochar (H_2_SO_4_)	SSA: 105.9; PV: 0.062; PS: 2.357; AC: 12.100	
13.	pristine	potato peel, pyrolysis temp. 550 °C	PV: 0.025; pH: 9.24; EC: 2.1; CEC: 57.16; AS: 12.06	[179]
	modified	CH_4_N_2_S-modified biochar	PV: 0.073; pH: 9.67; EC: 2.3; CEC: 74.39; AS: 13.18	
14.	pristine	waste eucalyptus chips, pyrolysis temp. 500 °C	SSA: 1265.56; PV: 1.31; PS: 3.91	[180]
	modified	Phosphoric-acid-activated biochar	SSA: 1654.38; PV: 1.85; PS: 4.12	
15.	pristine	pinecone biomass	SSA: 0.58; PV:0.001; PS: 7.40	[181]
	modified	biochar modification with NaOH or ZnCl_2_ (1/2 weight ratio of biochar/activator), drying, heating at 700–800 °C	SSA: 1470.27; PV: 0.705; PS: 2.96 SSA: 1067.90; PV: 0.511; PS: 2.68	
16.	pristine	rice straw, pyrolysis temp. 550 °C	SSA: 71.35	[150]
	modified	activation with KOH, HNO_3_, H_2_SO_4_, H_2_O_2_, KMnO_4_, activation conditions depended on the activator	SSA: 143.3; 87.2; 56.9; 110.9; 87.7, respectively	

Abbreviations: SSA is specific surface area (m^2^/g); PV is pore volume (cm^3^/g); PS is pore size (nm); AC is acidity (mmol/g); AS is ash (%); VM is volatile matter (%); EC is electrical conductivity (dS/m); and CEC is cation exchange capacity (cmol/kg).

## Data Availability

Not applicable.
